# Association of remnant cholesterol and lipid parameters with new-onset carotid plaque in Chinese population

**DOI:** 10.3389/fcvm.2022.903390

**Published:** 2022-08-30

**Authors:** Bo Liu, Fangfang Fan, Bo Zheng, Ying Yang, Jia Jia, Pengfei Sun, Yimeng Jiang, Kaiyin Li, Jiahui Liu, Chuyun Chen, Jianping Li, Yan Zhang, Yong Huo

**Affiliations:** ^1^Department of Cardiology, Peking University First Hospital, Beijing, China; ^2^Institute of Cardiovascular Disease, Peking University First Hospital, Beijing, China; ^3^Echocardiography Core Lab, Institute of Cardiovascular Disease, Peking University First Hospital, Beijing, China

**Keywords:** remnant cholesterol, carotid plaque, lipids, atherosclerosis, population study

## Abstract

**Background:**

Remnant lipoprotein cholesterol (RC) is an independent risk factor for cardiovascular disease (CVD). However, the relationships of remnant cholesterol and other conventional lipid parameters with new-onset carotid plaque are not fully understood in the Chinese community-based population.

**Materials and methods:**

A total of 872 plaque-free participants (51.39 ± 4.96 years old) with no history of CVD were included in this study. The plasma concentrations of RC were calculated by subtracting low-density lipoprotein cholesterol (LDL-C) and high-density lipoprotein cholesterol (HDL-C) from total cholesterol (TC). Multivariate regression models were used to evaluate and compare the associations between RC and other lipid parameters and new-onset carotid plaque.

**Results:**

After a mean 6.77-year follow-up, the incidence of new-onset carotid plaque was 188 (21.56%). RC was significantly associated with new-onset carotid plaque [Odd ratio (OR) = 1.57 per 1 mmol/L increase, 95% confidence interval (CI): 1.03–2.41, *p* = 0.038]. The highest tertile of RC (T3 group) had the highest risk of new-onset carotid plaque (*OR* = 2.53, 95% CI: 1.63–3.95). Similar results were seen for increased other lipid parameters, but decreased HDL-C levels. When adding another lipid parameter into the adjusted model with RC simultaneously, only RC remained significantly associated with new-onset carotid plaque after adjusting for other lipid parameters (all *p* value < 0.005). Furthermore, RC was strongly associated with new-onset carotid plaque in participants with lower baseline LDL-C levels.

**Conclusion:**

Increased RC levels were superior to other conventional lipid parameters to be associated with new-onset carotid plaque in the Chinese community-based population. Furthermore, RC should be considered in participants with lower LDL-C levels for the purpose of early atherosclerosis prevention.

## Introduction

There is considerable residual risk of arteriosclerotic cardiovascular disease (ASCVD) after reduction of low-density lipoprotein cholesterol (LDL-C) to the recommended concentration achieved by statin regimens, and even after managing other modifiable risk factors, such as hypertension (1–3). Over the past many years, numerous clinical studies have focused on high levels of triglyceride-rich lipoproteins cholesterol, which indicates increased concentrations of potentially remnant cholesterol and may help to explain the residual risk (4–6).

Remnant cholesterol is the cholesterol content of triglyceride-rich lipoproteins, and is composed of VLDL and IDL in the fasting state, and chylomicron remnants in the non-fasting state (2). When there is an excess of remnant lipoproteins in the plasma, remnants can carry large amounts of cholesterol and have the same potential ability as LDL to penetrate and become trapped in the intima of the arterial wall, resulting in the formation of foam cells, atherosclerosis, and low-grade inflammation (7–12).

The presence of new-onset carotid plaque frequently serves as a risk predictor in the assessment of CVD/Stroke risk, and carotid plaque formation is a surrogate marker of a high-risk of carotid atherosclerotic disease (13–15). The relationship between remnant lipoproteins cholesterol and cardiovascular events has been demonstrated for decades (4, 8, 16–20). However, few studies have focused on comparing the differences between RC and other conventional lipid parameters in atherosclerotic disease, even other surrogate markers, such as carotid plaque formation (21–24). In other words, the lack of development in the evidence base for the associations between RC and conventional lipid parameters and the risk of new-onset carotid plaque has been more important, especially in the Chinese community-based population with no history of cardiovascular disease (25).

The present study aimed to longitudinally evaluate the relationships between RC and other conventional lipid parameters and new-onset carotid plaque, and further assess the comparisons of RC and other parameters in relation to new-onset carotid plaque when both lipids were put into the model simultaneously.

## Materials and methods

### Study population

All participants included in this study were enrolled from a community-based atherosclerosis cohort set up in 2011 in Beijing, China. Detailed descriptions of the study procedures have been described previously (26). Initially, a total of 4,431 participants aged ≥ 40 years underwent the baseline survey in 2012 and responded on-site during the follow-up visit in 2018. For the present study, 1,960 participants with carotid plaque-free status at baseline were selected, and then 988 participants with quantitative carotid artery measurements at the follow-up visit were included. After stepwise exclusion, 116 participants included using lipid-lowering medications (*n* = 80), history of cardiovascular disease (*n* = 33), and missing data for lipid profiles (*n* = 3). Ultimately, this analysis included 872 eligible participants with a mean 6.77-year follow-up (Supplementary Figure 1). This study was approved by the ethics committee of Peking University First Hospital, and confirmed to the provisions of the Declaration of Helsinki. All participants signed informed consent.

### Data collection

Baseline and follow-up data were collected by trained researcher staff according to standard operating procedures. All participants were interviewed using a standard questionnaire that was specifically designed for the present study, to obtain information on demographic characteristics, education, occupation, lifestyle, personal, and medical history. Current smoking means smoking at least one cigarette per day for at least 6 months. Current drinking means drinking alcohol at least once per week for at least 6 months. The body mass index (BMI) was calculated by the following formula: BMI = weight (kg)/height (m^2^). The peripheral systolic (SBP) and diastolic blood pressure (DBP) readings used the mean value of these three successful measurements using a standard method (26).

A venous blood sample was obtained from the forearm of each participant after an overnight fast (at least 12 h) at the baseline survey. Subsequently, the Roche C8000 Automatic Analyzer was used to examine all biochemistry parameters in serum, including fasting blood glucose (FBG), 2-h glucose in the standard 75-g oral glucose tolerance test (OGTT), total cholesterol (TC), triglycerides (TG), high-density lipoprotein cholesterol (HDL-C), and low-density lipoprotein cholesterol (LDL-C), which were also directly measured by a chemical method; serum creatinine (Scr, μmol/L) levels were measured enzymatically. Non-HDL-C was calculated by subtracting HDL-C from TC. RC was calculated by subtracting LDL-C and HDL-C from TC, as done previously (21, 27, 28). In addition, the estimated glomerular filtration rate (eGFR) was determined by the CKD-EPI equation (26).

Hypertension was defined as any self-reported history, SBP ≥ 140 mmHg or DBP ≥ 90 mmHg, or taking anti-hypertensive medication. Diabetes mellitus was defined as any self-reported history of diabetes, use of hypoglycemic medication, FBG ≥ 7.0 mmol/L, and/or OGTT ≥ 11.1 mmol/L.

### Carotid ultrasonography

All participants underwent carotid ultrasonography by trained and certified sonographers both at the baseline survey in 2012 using the high-resolution B-mode ultrasound system (GE Vivid 7, 8∼10 MHz linear-array vascular transducer; Milwaukee, WI, United States) and at the follow-up visit in 2018 using a Terason Echo Ultrasound System (Burlington, MA, United States). Briefly, carotid ultrasound was performed according to standard scanning and reading protocols at the baseline survey and follow-up visit. Intima-media thickness (IMT) was detected as the distance between the lumen-intima and the media-adventitia ultrasound interfaces. Carotid IMT (cIMT) was defined as the mean IMT measured at 1 cm lengths of the far wall of the bilateral distal common carotid artery. Carotid plaque was defined as focal structures encroaching into the arterial lumen of at least 0.5 mm or 50% of the surrounding cIMT value, or demonstrating a thickness > 1.5 mm as measured from the intima–lumen interface to the media-adventitia interface at any level of the bilateral common carotid artery, internal carotid artery, and/or bifurcation (29).

### Statistical analysis

Descriptive statistics were expressed as the mean ± standard deviation (SD) or median (interquartile range) for continuous variables and number (percentage) for dichotomous variables. Normally distributed continuous variables were compared using Student’s *t*-test, whereas Kruskal-Wallis test was used for variables with a skewed distribution. Pearson’s χ^2^-test or Fisher’s exact test was applied to all categorical variables as appropriate. Univariate and multivariate regression models were used to evaluate the relationships between baseline lipid parameters (both as a continuous and categorical variable) and new-onset carotid plaque, after adjusting for sex and age (Model 1), and further adjusting for BMI, current smoking, current drinking, estimated glomerular filtration rate, diabetes mellitus, hypertension, and the use of antihypertensive and hypoglycemic medications (Model 2). Regarding possible collinearity, the variance inflation factor (VIF) was calculated for the included variables in each multivariable regression model (Supplementary Table 1). We further assessed the comparisons of RC and other conventional lipid parameters in relation to new-onset carotid plaque when both lipid parameters were put into the model simultaneously. In addition, we conducted threshold effect analysis for lipid parameters if the relationships were non-linear (Supplementary Table 2), and investigated the modification of baseline LDL-C levels for the effect of RC on new-onset carotid plaque. In this study, a *P*-value of < 0.05 (two-sided) was considered statistically significant for all tests. All statistical analyses were performed using Empower(R) (X&Y solutions, Inc., Boston, MA, United States) and R software.^1^

## Results

### Baseline patient characteristics

Table 1 shows the baseline characteristics of eligible participants, both overall and stratified by RC tertiles. Among the 872 subjects, 73.62% were female, with an average age of 51.39 ± 4.96 years old and a mean (SD) BMI of 25.62 ± 3.31 kg/m^2^. Those with hypertension and diabetes accounted for 26.95% (235), and 12.96% (113), respectively. The mean (SD) baseline lipid parameters were 5.27 ± 0.90 mmol/L for TC, 3.20 ± 0.74 mmol/L for LDL-C, 1.49 ± 0.40 mmol/L for HDL-C, and 3.78 ± 0.91 mmol/L for non-HDL-C, respectively. The median (interquartile range, IQR) RC was 0.52 (0.37, 0.70)°mmol/, and TG was 1.22 (0.88, 1.77)°mmol/L. The participants with higher RC (the top tertile) had higher levels of BMI, TC, LDL-C, TG, non-HDL-C, FBG, lower levels of HDL-C, and a higher prevalence of hypertension, diabetes mellitus (*p* < 0.05). There was no significant difference between the different RC tertiles for current drinking, or the use of anti-hypertensive and hypoglycemic medication.

**TABLE 1:** Baseline characteristics stratified by remnant lipoprotein cholesterol (RC) tertiles.

	Total	Remnant cholesterol, mmol/L			*P-value*
		Tertile 1 (< 0.42)	Tertile 2 (0.42- < 0.64)	Tertile 3 (≥ 0.64)	
N	872	290	290	292	
Age, year	51.39 ± 4.96	50.86 ± 5.01	51.01 ± 5.11	52.30 ± 4.62	< 0.001
Female, N (%)	642 (73.62%)	227 (78.28%)	213 (73.45%)	202 (69.18%)	0.045
BMI, kg/m^2^	25.62 ± 3.31	24.35 ± 3.12	25.84 ± 3.25	26.67 ± 3.15	< 0.001
Total cholesterol, mmol/L	5.27 ± 0.90	4.83 ± 0.71	5.21 ± 0.80	5.76 ± 0.92	< 0.001
Triglycerides, mmol/L	1.22 (0.88, 1.77)	0.79 (0.63, 1.02)	1.23 (1.00, 1.55)	2.07 (1.54, 2.71)	< 0.001
HDL-C, mmol/L	1.49 ± 0.40	1.74 ± 0.41	1.46 ± 0.33	1.26 ± 0.29	< 0.001
LDL-C, mmol/L	3.20 ± 0.74	2.79 ± 0.57	3.23 ± 0.65	3.57 ± 0.78	< 0.001
Non-HDL-C, mmol/L	3.78 ± 0.91	3.09 ± 0.60	3.76 ± 0.66	4.50 ± 0.83	< 0.001
Remnant cholesterol, mmol/L	0.52 (0.37–0.70)	0.32 (0.24–0.37)	0.52 (0.47–0.58)	0.81 (0.70–0.97)	< 0.001
FBG, mmol/L	5.84 ± 1.49	5.64 ± 1.43	5.71 ± 1.05	6.17 ± 1.83	< 0.001
eGFR, mL/min/1.73°m^2^	100.23 ± 9.42	101.75 ± 8.82	101.03 ± 9.27	97.92 ± 9.73	< 0.001
Current drinking, N (%)	196 (22.48%)	57 (19.66%)	69 (23.79%)	70 (23.97%)	0.370
Current smoking, N (%)	133 (15.25%)	33 (11.38%)	41 (14.14%)	59 (20.21%)	0.010
**Disease, N (%)**					
Hypertension	235 (26.95%)	57 (19.66%)	67 (23.10%)	111 (38.01%)	< 0.001
Diabetes mellitus	113 (12.96%)	30 (10.34%)	28 (9.66%)	55 (18.84%)	0.001
**Treatment, N (%)**					
Antihypertensive	114 (13.07%)	35 (12.07%)	33 (11.38%)	46 (15.75%)	0.242
Hypoglycemic	34 (3.90%)	12 (4.15%)	7 (2.41%)	15 (5.14%)	0.229

Data are shown as mean ± standard deviation (SD) or median (IQR, Q1-Q3) for continuous variables and number (percentage) for dichotomous variables.

BMI, body mass index; RC, remnant cholesterol; TC, total cholesterol; TG, triglycerides; HDL-C, high-density lipoprotein cholesterol; LDL-C, low-density lipoprotein cholesterol; Non-HDL-C, non-high-density lipoprotein cholesterol; FBG, fasting blood glucose; eGFR, estimated glomerular filtration rate.

### Associations of remnant lipoprotein cholesterol and other lipid parameters with new-onset carotid plaque when considered individually

**FIGURE 1:**
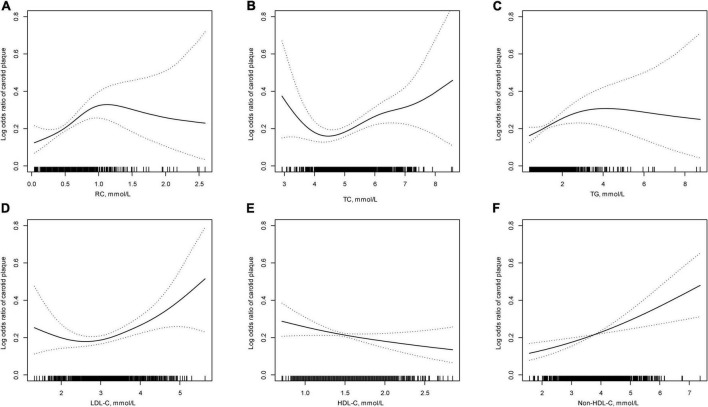
The relationship between lipid parameters and new-onset carotid plaque*. **(A)** Remnant lipoprotein cholesterol (RC); **(B)** total cholesterol (TC); **(C)** triglycerides (TG); **(D)** low-density lipoprotein cholesterol (LDL-C); **(E)** high-density lipoprotein cholesterol (HDL-C); and **(F)** Non-HDL-C. *Adjusted for: sex, age, body mass index, current drinking, current smoking, estimated glomerular filtration rate, diabetes mellitus, hypertension, antihypertensive, and hypoglycemic drugs.

Of the 872 eligible plaque-free participants at baseline in this study, 188 (21.56%) individuals developed new-onset carotid plaque after a mean 6.77-year follow-up. As shown in Figure 1, there was mainly positive association between lipid parameters and new-onset carotid plaque, except for a negative linear association with HDL-C. Table 2 demonstrates the associations of RC and other conventional lipid parameters with new-onset carotid plaque. RC (per 1 mmol/L increase) was significantly associated with increases of 65% (95% CI: 1.10–2.48; *p* = 0.016) for the risk of new-onset carotid plaque. In the adjusted multivariable regression models, increased RC was strongly associated with new-onset carotid plaque (*OR* = 1.57 per 1 mmol/L increase; 95% CI: 1.03–2.41; *p* = 0.038). Similar results appeared in lipid parameters as categorical variables in tertiles, and showed a gradient relationship except for HDL-C (*p* for trend < 0.05).

**TABLE 2:** Logistic regressions for the effects of baseline lipid parameters and new-onset carotid plaque.

Lipid parameters	N (%)	OR (95% CI) *P-value*		
		Crude	Adjusted model 1	Adjusted model 2
RC, per 1 mmol/L increase	188 (21.56%)	1.65 (1.10–2.48) 0.016	1.52 (1.02–2.28) 0.042	1.57 (1.03–2.41) 0.038
**Tertiles of RC**				
T1 (< 0.42)	46 (15.86%)	Ref.	Ref.	Ref.
T2 (0.42–< 0.64)	50 (17.24%)	1.11 (0.71–1.71) 0.655	1.07 (0.69–1.67) 0.764	1.23 (0.78–1.95) 0.376
T3 (≥ 0.64)	92 (31.51%)	2.44 (1.64–3.64) < 0.001	2.18 (1.45–3.28) < 0.001	2.53 (1.63–3.95) < 0.001
*p* for trend		< 0.001	< 0.001	< 0.001
TC, per 1 mmol/L increase	188 (21.56%)	1.28 (1.08–1.54) 0.006	1.30 (1.08–1.56) 0.006	1.28 (1.06–1.55) 0.011
**Tertiles of TC**				
T1 (< 4.87)	50 (17.30%)	Ref.	Ref.	Ref.
T2 (4.87–< 5.60)	59 (20.21%)	1.21 (0.80–1.84) 0.370	1.25 (0.81–1.91) 0.313	1.23 (0.79–1.90) 0.355
T3 (≥ 5.60)	79 (27.15%)	1.78 (1.19–2.66) 0.005	1.81 (1.19–2.76) 0.005	1.81 (1.18–2.78) 0.007
*p* for trend		0.004	0.005	0.006
TG, per 1 mmol/L increase	188 (21.56%)	1.15 (1.01–1.31) 0.040	1.12 (0.97–1.28) 0.115	1.12 (0.97–1.30) 0.128
**Tertiles of TG**				
T1 (< 0.99)	45 (15.68%)	Ref.	Ref.	Ref.
T2 (0.99–< 1.55)	64 (21.92%)	1.51 (0.99–2.30) 0.056	1.49 (0.97–2.29) 0.070	1.55 (0.99–2.41) 0.053
T3 (≥ 1.55)	79 (26.96%)	1.99 (1.32–2.99) 0.001	1.79 (1.18–2.72) 0.006	1.92 (1.22–3.00) 0.004
*p* for trend		0.001	0.007	0.005
HDL-C, per 1 mmol/L increase	188 (21.56%)	0.63 (0.41–0.96) 0.032	0.73 (0.46–1.15) 0.175	0.64 (0.39–1.05) 0.079
**Tertiles of HDL-C**				
T1 (< 1.28)	69 (23.88%)	Ref.	Ref.	Ref.
T2 (1.28–< 1.60)	69 (23.79%)	1.00 (0.68–1.46) 0.982	1.03 (0.69–1.55) 0.867	0.96 (0.64–1.46) 0.863
T3 (≥ 1.60)	50 (17.06%)	0.66 (0.44–0.99) 0.042	0.74 (0.48–1.15) 0.183	0.65 (0.41–1.05) 0.077
*p* for trend		0.046	0.188	0.0780
LDL-C, per 1 mmol/L increase	188 (21.56%)	1.43 (1.15–1.78) 0.001	1.40 (1.12–1.76) 0.003	1.40 (1.11–1.77) 0.004
**Tertiles of LDL-C**				
T1 (< 2.85)	50 (17.42%)	Ref.	Ref.	Ref.
T2 (2.85–< 3.46)	58 (19.80%)	1.17 (0.77–1.78) 0.463	1.10 (0.72–1.69) 0.666	1.14 (0.73–1.76) 0.567
T3 (≥ 3.46)	80 (27.40%)	1.79 (1.20–2.67) 0.004	1.70 (1.13–2.57) 0.012	1.75 (1.14–2.67) 0.010
*p* for trend		0.004	0.010	0.009
Non-HDL-C, per 1 mmol/L increase	188 (21.56%)	1.39 (1.16–1.65) < 0.001	1.35 (1.12–1.61) 0.001	1.36 (1.13–1.65) 0.001
**Tertiles of Non-HDL-C**				
T1 (< 3.37)	49 (16.96%)	Ref.	Ref.	Ref.
T2 (3.37–< 4.10)	57 (19.52%)	1.19 (0.78–1.81) 0.424	1.09 (0.71–1.68) 0.696	1.14 (0.73–1.77) 0.571
T3 (≥ 4.10)	82 (28.18%)	1.92 (1.29–2.87) 0.001	1.81 (1.20–2.73) 0.005	1.89 (1.23–2.89) 0.004
*p* for trend		0.001	0.003	0.003

Model 1: adjusted for age and sex. Model 2: adjusted for age, sex, body mass index, current drinking, current smoking, estimated glomerular filtration rate, diabetes mellitus, hypertension, antihypertensive, and hypoglycemic drugs.

OR, odds ratio; CI, confidence interval; Ref., reference value; RC, remnant cholesterol; TC, total cholesterol; TG, triglycerides; HDL-C, high-density lipoprotein cholesterol; LDL-C, low-density lipoprotein cholesterol; Non-HDL-C, non-high-density lipoprotein cholesterol.

### Associations of remnant lipoprotein cholesterol and other lipid parameters with new-onset carotid plaque when considered simultaneously

When RC and another conventional lipid parameter were put into the multivariable regression model simultaneously, only RC remained significantly associated with new-onset carotid plaque, even after adjusting for other lipid parameters respectively in different comparisons. Compared with the bottom tertile (T1), the effect of higher RC (the top tertile) for new-onset carotid plaque was increased by 2.26 (95% CI: 1.40–3.65) after adjusting for TC, 2.55 (95% CI: 1.41–4.16) after adjusting for TG, 2.54 (95% CI: 1.55–4.15) after adjusting for HDL-C, 2.25 (95% CI: 1.39–3.63) after adjusting for LDL-C, and 2.28 (95% CI: 1.31–3.97) after adjusting for non-HDL-C, respectively (Table 3).

**TABLE 3:** Comparisons of remnant lipoprotein cholesterol (RC) and another lipid parameter in relation to new-onset carotid plaque.

Comparisons	OR (95% CI) *P-value*		OR (95% CI) *P-value*
**Comparison I^†^ (when considered RC and TC simultaneously)**			
**RC, mmol/L**		**TC, mmol/L**	
**T1 (< 0.42)**	**Ref.**	**T1 (< 4.87)**	**Ref.**
T2 (0.42–< 0.64)	1.16 (0.73–1.86) 0.525	T2 (4.87–< 5.60)	1.12 (0.72–1.75) 0.611
T3 (≥ 0.64)	2.26 (1.40–3.65) < 0.001	T3 (≥ 5.60)	1.33 (0.83–2.12) 0.230
**Comparison II^†^ (when considered RC and TG simultaneously)**			
**RC, mmol/L**		**TG, mmol/L**	
**T1 (< 0.42)**	**Ref.**	**T1 (< 0.99)**	**Ref.**
T2 (0.42–< 0.64)	1.16 (0.70–1.94) 0.564	T2 (0.99–< 1.55)	1.26 (0.77–2.07) 0.363
T3 (≥ 0.64)	2.55 (1.41–4.61) 0.002	T3 (≥ 1.55)	1.01 (0.55–1.85) 0.963
**Comparison III^†^ (when considered RC and HDL-C simultaneously)**			
**RC, mmol/L**		**HDL-C, mmol/L**	
**T1 (< 0.42)**	**Ref.**	**T1 (< 1.28)**	**Ref.**
T2 (0.42–< 0.64)	1.21 (0.75–1.94) 0.428	T2 (1.28–< 1.60)	1.22 (0.79–1.88) 0.377
T3 (≥ 0.64)	2.54 (1.55–4.15) < 0.001	T3 (≥ 1.60)	1.02 (0.60–1.74) 0.929
**Comparison IV^†^ (when considered RC and LDL-C simultaneously)**			
**RC-mmol/L**		**LDL-C, mmol/L**	
**T1 (< 0.42)**	**Ref.**	**T1 (< 2.85)**	**Ref.**
T2 (0.42–< 0.64)	1.14 (0.71–1.84) 0.578	T2 (2.85–< 3.46)	1.07 (0.68–1.67) 0.772
T3 (≥ 0.64)	2.25 (1.39–3.63) < 0.001	T3 (≥ 3.46)	1.33 (0.84–2.11) 0.230
**Comparison V^†^ (when considered RC and Non-HDL-C simultaneously)**			
**RC, mmol/L**		**Non-HDL-C, mmol/L**	
**T1 (< 0.42)**	**Ref.**	**T1 (< 3.37)**	**Ref.**
T2 (0.42–< 0.64)	1.19 (0.72–1.96) 0.494	T2 (3.37–< 4.10)	0.94 (0.59–1.52) 0.811
T3 (≥ 0.64)	2.28 (1.31–3.97) 0.004	T3 (≥ 4.10)	1.17 (0.68–2.00) 0.569

^†^RC and other lipid parameters were simultaneously added into the multivariable regression model. The model was adjusted for age, sex, body mass index, current drinking, current smoking, estimated glomerular filtration rate, diabetes mellitus, hypertension, antihypertensive, and hypoglycemic drugs.

OR, odds ratio; CI, confidence interval; Ref., reference value; RC, remnant cholesterol; TC, total cholesterol; TG, triglycerides; HDL-C, high-density lipoprotein cholesterol; LDL-C, low-density lipoprotein cholesterol; Non-HDL-C, non-high-density lipoprotein cholesterol.

### Association of remnant lipoprotein cholesterol for new-onset carotid plaque modified by baseline low-density lipoprotein cholesterol levels

**FIGURE 2:**
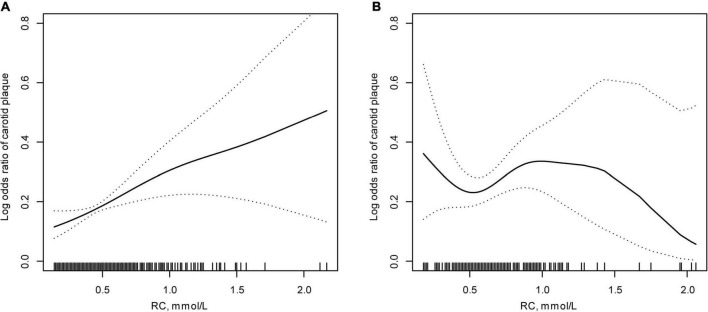
Effect of new-onset carotid plaque based on remnant lipoprotein cholesterol (RC) modified by low-density lipoprotein cholesterol (LDL-C) levels*. **(A)** Baseline LDL-C < 3.4 mmol/L; **(B)** baseline LDL-C ≥ 3.4 mmol/L. *Adjusted for: sex, age, body mass index, current drinking, current smoking, estimated glomerular filtration rate, diabetes mellitus, hypertension, antihypertensive, and hypoglycemic drugs.

Furthermore, we investigated the modification of baseline LDL-C levels for the effect of RC on new-onset carotid plaque in participants with baseline LDL-C levels. After adjusting for possible covariates, Figure 2 displays the smooth curves showing the relationships between RC and new-onset carotid plaque stratified by baseline LDL-C. Table 4 shows that baseline LDL-C levels modified the association of RC for new-onset carotid plaque, with an increased OR to 1.95 (95% CI: 1.06–3.56) in participants with lower baseline LDL-C levels (*p* for interaction = 0.044).

**TABLE 4:** Association of remnant lipoprotein cholesterol (RC) for new-onset carotid plaque modified by baseline low-density lipoprotein cholesterol (LDL-C) levels.

Variables	N (%)	OR (95% CI)	*P-value*	*p* interaction
**LDL-C, mmol/L**				
< 3.4	105 (18.72%)	1.95 (1.06–3.56)	0.031	0.044
≥ 3.4	83 (26.69%)	0.97 (0.40–2.34)	0.952	

Model adjusted for age, sex, body mass index, current drinking, current smoking, estimated glomerular filtration rate, diabetes mellitus, hypertension, antihypertensive, and hypoglycemic drugs.

OR, odds ratio; CI, confidence interval; LDL-C, low-density lipoprotein cholesterol.

## Discussion

The major findings of this study are that conventional lipid parameters, especially RC, were superiorly associated with new-onset carotid plaque, independent of other lipids, in Chinese community-based population with no history of cardiovascular disease. Additionally, among participants with lower baseline LDL-C levels, RC should be considered an important biomarker to assess carotid artery atherosclerosis risk.

Previous studies have already investigated the relationship between remnant lipoprotein cholesterol and cardiovascular diseases (8, 16–20, 30–34). Remnant cholesterol was considered a risk factor for various cardiovascular events. Varbo and colleagues found that elevated RC could causes ischemic heart disease, independent of reduced HDL-C (8). Remnant-like particle (RLP) cholesterol has also similarly been shown to be an independent risk factor for cardiovascular disease among 1,567 women from the Framingham Heart Study (30), and in elderly Japanese coronary artery disease (CHD) patients (31). In addition, some prospective studies have been presented supporting the prognostic value of remnant lipoprotein for cardiovascular disease, the results from the Jackson Heart Study and Framingham Offspring Cohort Study demonstrated that RC was positively associated with incident CHD events, but the association was not significant after adjustments for HDL-C and LDL-C (16). Some studies have reported the significant association between remnant lipoprotein cholesterol and the risk of coronary events in CHD or ACS patients with or without diabetes (32–36).

However, few studies have focused on carotid atherosclerosis assessed by carotid plaque. Masson et al. conducted a cross-sectional study and concluded that higher RC was associated with the presence of carotid atherosclerotic plaque (21). In the present study, a superior independent association of increased RC levels with new-onset carotid plaque compared to other conventional lipid parameters was demonstrated.

Several potential mechanisms may account for the effect of elevated levels of RC on new-onset carotid plaque. Like LDL-C passing the endothelial layer and trapping into the arterial intima, this would lead to the accumulation of cholesterol, the occurrence of atherosclerosis and cardiovascular events (3). Unlike LDL, remnant lipoprotein cholesterol could be taken up directly (no need to be modified: oxidation) by macrophages to cause foam cell formation and atherosclerotic plaque formation (37). Additionally, it has been shown that RC is an indicator of endothelial vasomotor dysfunction (38) that can upregulate the expression of pro-inflammatory factors (facilitate monocyte movement into the arterial wall), adhesion molecules (promote the formation of thrombus) (39), and coagulation factors (enhance the aggregation of platelets) (40). Elevated RC was causally associated with low-grade inflammation at a whole-body level, with 37% higher C-reactive protein levels for 1-mmol/L higher levels of RC (12), and related to carotid macrophage content, a marker for plaque instability (24). Taken together, the direct and indirect roles (pro-inflammatory and pro-atherothrombotic) of remnant lipoprotein cholesterol could partially explain increased risk of new-onset carotid plaque.

In addition, Nakamura et al. reported that RC was superior to non-HDL-C for predicting cardiovascular events with LDL-C levels < 2.6 mmol/L treated with statins in patients with coronary artery disease (41). Consistently, our study demonstrated that increased RC levels were more strongly associated with the risk of new-onset carotid plaque when comparing two lipid parameters in the same model simultaneously. Studies have reported that increased RC can explain part of the residual risk of cardiovascular disease with lower or well-controlled levels of LDL-C goal (4, 42). Lin et al. found that higher RC concentrations were significantly associated with coronary atherosclerotic burden, even with an optimal level of LDL-C (23). In another study, the investigator reported that subjects with higher baseline RC had a higher risk of major adverse cardiovascular events (MACEs) than those at lower concentrations, especially lower LDL-C levels, with a highest HR of 2.69 (*p* = 0.001) (20). Similarly, our study found that the stronger association of RC with the risk of new-onset carotid plaque was demonstrated in participants with lower baseline LDL-C levels (< 3.4 mmol/L), which indicated that RC remained a residual risk factor for ASCVD for new-onset carotid plaque when LDL-C achieved to goal (< 3.4 mmol/L). A similar study demonstrated that the high RC/low LDL-C group, was associated with increased ASCVD risk (43).

The present study, to the best of our knowledge, is the first to evaluate the associations between RC and new-onset carotid plaque, and to compare RC and other lipid parameters in relation to new-onset carotid plaque in the Chinese population. Additionally, different baseline LDL-C levels modified the association of RC for carotid plaque. There are several limitations that need to be addressed. First, all participants were from a community-based cohort, and therefore external generalizability is limited. Second, the use of fasting samples may underestimate the contribution of chylomicron, due to VLDL are the dominant constituents of circulating remnants (44), and calculated RC cannot be as accurate as direct measurement, while it’s easier to calculate RC by other conventional lipid parameters to save costs, and the association was remarkably consistent (27, 45–47). Third, data such as inflammatory biomarkers, dietary habits, fatty liver, vascular ultrasound in other territories, etc., were not collected at baseline, which may affect atherosclerosis formation. Finally, carotid plaque formation is a marker for carotid artery damage to evaluate the risk of cardiovascular events, and the need to observe the risk of MACEs during continuous follow-up should be considered.

In conclusion, remnant cholesterol was superior and independent of other conventional lipid parameters, and was significantly associated with new-onset carotid plaque when considered simultaneously. Remnant cholesterol could be helpful to predict carotid artery damage in participants with lower baseline LDL-C levels for the purpose of early atherosclerosis prevention.

## Data availability statement

The raw data supporting the conclusions of this article will be made available by the authors, without undue reservation.

## Ethics statement

The studies involving human participants were reviewed and approved by Peking University First Hospital Ethics Committee. The patients/participants provided their written informed consent to participate in this study.

## Author contributions

YZ and YH were responsible for the study concept and design. BZ, YY, and JL helped with the design and coordination of the study. PS, YJ, KL, JhL, and CC collected and rechecked the data. FF, JJ, and BL analyzed and interpreted the data. BL drafted the manuscript. FF and YZ revised the manuscript. All authors reviewed and approved the manuscript and agreed to be accountable for all aspects of the work.

## Footnotes

1
www.R-project.org
