# Supplementary material for: Association of remnant cholesterol and lipid parameters with new-onset carotid plaque in Chinese population
Source: Front Cardiovasc Med. 2022 Aug 30;9:903390. doi: 10.3389/fcvm.2022.903390 (PMC9468421; doi:10.3389/fcvm.2022.903390)
Supplement: Supplementary file 1 [file Table_1.DOCX]

Supplementary Material

**Supplementary Figure 1.** Flow chart of study participants

# Supplementary Table 1. Stepwise collinear screening for included variables in the adjusted model

| **Variables** | **VIF** |
| --- | --- |
| Remnant cholesterol | 1.1 |
| Sex | 2 |
| Age | 1.3 |
| Body mass index | 1.1 |
| Current drinking | 1.5 |
| Current smoking | 1.7 |
| eGFR | 1.3 |
| Hypertension | 1.8 |
| Antihypertensive | 1.7 |
| Diabetes mellitus | 1.4 |
| Hypoglycemic | 1.4 |

Abbreviations: eGFR, estimated glomerular filtration rate.

# Supplementary Table 2. Threshold effect analysis of RC on new-onset plaque using piecewise linear regression model

| **Model** | **Result [OR* (95%CI)]** | **p value** |
| --- | --- | --- |
| Model 1 one-line | 1.57 (1.03-2.41) | 0.038 |
| Model 2 turning point: 0.91 mmol/L | | |
| Slope 1: <0.91 mmol/L | 5.78 (2.43-13.74) | <0.001 |
| Slope 2: ≥0.91 mmol/L | 0.51 (0.19-1.41) | 0.196 |
| Slope 2 - Slope 1 | 0.09 (0.02-0.41) | 0.002 |
| A log likelihood ratio test |  | <0.001 |

*OR, odds ratio, represented the effect for per 1mmol/L increase of RC. Adjusted for age, sex, body mass index, current drinking, current smoking, estimated glomerular filtration rate, diabetes mellitus, hypertension, antihypertensive and hypoglycemic drugs.

Abbreviations: OR, odds ratio; CI, confidence interval; RC, remnant cholesterol.
